# Account of a Cæsarean Operation through the Vagina, Successfully Performed

**Published:** 1817-05

**Authors:** M. Grimme

**Affiliations:** Aulic Surgeon and Man-Midwife at Brunswig.


*For the London Medical and Physical Journal.*

*Account of a Cæsarean Operation through the Vagina, successfully performed;* by M. GRIMME, Aulic Surgeon and Man-Midwife at Brunswig. Communicated by Dr. VON EMBDEN.

IN the night of the 22d of November, 1814, Mr. G. was called to assist the wife of a shoemaker of that place, the midwife having declared it impossible to deliver her. After several strange details the midwife gave of the case, as, for instance, that the whole uterus had prolapsed, that the os tincæ had totally disappeared, that absolutely no part of the child could be felt, and that the patient had felt labour-pains sixteen hours already, &c. Mr. G. proceeded to examine, and found the abdomen regularly shaped. The waters had not yet come away, and the movements of the child was plainly perceivable in the upper and right side of the uterus. The woman was twenty-nine years of age, of an ordinary size, and the pelvis well proportioned. On examining the pudenda, however, the left labium was found swelled as big as a man's head, the right being of the proper size. Irregular flaps, and pieces of flesh, made up the lesser labia; and the whole entrance of the vagina appeared lacerated, though not sore, all being healed, and shewing only that inflammation and suppuration had once existed in those parts. Of this Mr. G. afterwards actually convinced himself, being told, that, twelve years ago, after a lying-in in Swedish Pomerania, the patient had laboured a whole year under an affection of these parts, and had actually undergone surgical treatment. The clitoris appeared also lacerated, and in the vagina scars were plainly seen; so that there was every reason to suppose the womb at that time had participated in the inflammation and subsequent suppuration, and that the vaginal portion leading to it had been destroyed. A tumour was felt in the upper part of the vagina, occupying the whole upper pelvic aperture, the right vaginal portion lying extended before it, and feeling like an amnion ready to burst. Passing his finger a little beyond that vaulted swelling, backward and to the left, an aperture was discovered like a small grove: this could be nothing but the disfigured remains of an os tincæ. This little aperture had hitherto entirely escaped the observation of the midwife, as, during the frequent and violent pains, the examining finger could but with difficulty be carried thither, and during the intervals it could not be reached at all. In the front of the fundus vaginæ, several uneven spots were were met with, very probably scars of healed-up lacerations and wounds: these uneven places were, however, removed, and felt quite smooth, by the protrusion of the large vaulted tumour containing the child, and the liquor amnii enveloping it. On that side, the examining fingers were daubed by streaks of blood issuing from that small aperture in the left fundus vaginæ, rendering it still more certain that the os tincæ was here situated, and that the tumour descending into the pelvis was formed by the upper portion of the vagina and the right lateral portion of the uterus, lying close to the os tincæ. Mr. G. directed the patient to assume a left lateral posture, and lifted the abdomen a little, pressing towards that side, without being, however, able to produce any alteration; each pain throwing the patient again on her back, the throes being so violent as to render the abdomen uncommonly hard, the face flushed and swelled, and the neck, with all its visible vessels, much distended.

The protruding of the external pudenda during each pain, the pretty plain protrusion of the child's head and of the liquor amnii into the middle aperture of the pelvis, yet without any visible possibility of the child being born that way, suggested to Mr. G. the making an incision into the fundus vaginæ, beginning from the little aperture mentioned before. In doing this, he thought himself the more justified, as, from a consideration of all circumstances taken together, there was no reason to suppose an extra-uterine fœtus.

Mr. G. wishing for some assistance, Dr. Sagehorn, formerly also in the habit of practising midwifery, was consulted; and Mr. G. himself being under the necessity of going for his instruments, brought with him M. Berger, Prosector at the Anatomical Theatre.

They assembled in about an hour, and found the patient's condition so changed, that the little grove on the left side of the vaulted protrusion of the vagina had become a triangular hole, each margin of which, perhaps, might have amounted to half an inch; and that more blood, of a bright colour, issued from the vagina. The labour-pains, the midwife reported, had, in the mean time, been very strong and violent. The patient was still suffering greatly. The pulse was full, hard, irregular, sometimes quick, at others slow; in short, her state was such as made speedy assistance absolutely necessary. Both practitioners agreeing with Mr. G. as to the method he proposed, and the chair of delivery at hand being too low for a couch, the patient's bed was adapted for the purpose.

In consequence of the severe pains, which now hardly ever ceased, the vaulted tumour kept descending lower down in the the pelvis; the aperture on its left side became somewhat larger, in consequence of the laceration of the fibres, which was discovered by some florid blood issuing forth along the examining finger: this aperture could, however, now only be reached during the remission of the pains.

Mr. G. now introduced four fingers of his left hand into the vagina, in such a manner as to bring the symphysis ossium pubis between them and the thumb; took a headed bistoury with a handle (the fistula knife of Savigne); directed the head between the fingers up into the small aperture, situated upwards to the left in the pelvis, dividing, with the edge of the knife, the right side of the womb, about an inch and a half to two inches; and, shifting his hand, or rather the points of his fingers, in the same moment, through that aperture, he discovered them to be on the interior surface of the uterus. The amnion now pressed forward into this artificial os tincæ in the usual manner, the right side, however, presenting the unlimited and smooth internal surface of the uterus.

Mr. G. now enlarged the first incision by half an inch towards the right, and, the edges being so thin as to threaten laceration, he was induced to make two more small incisions, backward and towards the right, as also one in front. Immediately the amnion burst, a considerable quantity of liquor amnii was discharged, and the head of the child entered the aperture newly formed, in a perfectly regular manner. The right portion of the uterus now kept pressing down in the pelvis, along with the larger part of the child's head. The small remains of the natural os tincæ, or rather its vestige, could now no longer be reached on the left side, and the edges of the artificial one were so slender that they were hardly felt.

The os tincæ thus offered, on the left side, no obstacle to the passage of the head; only the right portion of the womb could not be made to glide over its thicker part, though its greatest part had already descended into the lesser pelvis. Under these circumstances, Mr. G. hesitated whether he should still enlarge the incision to the right. Having only that portion of the womb to work upon, he felt very reluctant to divide it. Thus he resolved to apply the forceps, not, indeed, for the purpose of evolving the head, but rather, if possible, to support it a little, in order to be able to await the distension of the divided womb, which, at all events, he considered necessary. The labour-pains, and the consequent protrusion of the head, were so violent, as to make the laceration of the fibres of the uterus appear unavoidable, which Mr. G. now thought it prudent to prevent, by another incision cision to the right, of about an inch, considering it not likely to produce any great danger. This being done, the child's head appeared in the external pudenda uncovered by the womb. But now another impediment occurred, on the left side, at the outlet of the vagina, namely, a stricture, most probably a cicatrix, that did not admit sufficient room for the head to pass through. Mr. G. having both his hands engaged, the right in conducting the handle of the forceps, and the left in supporting the perineum; Mr. Berger, standing to his left and to the right of the patient, took a bistoury curved like a sabre, and divided the callous part; whereupon the head evolved spontaneously, without requiring the least assistance.

It is worth remarking, that the child cried aloud and respired as soon as the head was protruded, though the chest was yet entirely hidden in the vagina; as also, that the one side of the uterus, (namely, the portion lying next to the os tincæ,) offered every distension to the passage of the child, whilst the left was not distended, though the fundus uteri did not incline to any side, as is sometimes the case. Nevertheless, Mr. G. had the uterus moved towards the left during the operation, and made the patient recline to that side. As long as the operation lasted, and during the various incisions, little blood had flown, and but a moderate quantity came away during the expulsion of the placenta, which happened in about five minutes afterwards. The patient hardly felt the first incision, but the latter lesser ones gave her great uneasiness, of which she complained each time. When the delivery was accomplished, the patient felt very weak; she slept an hour soon after; and, on Mr. G.'s visiting her about five minutes later, he found her in a gentle perspiration, her pulse soft and slow, and she felt some inclination to take refreshment. During the first three days, she complained of no other uneasiness than the after-pains, and of these only when she moved herself, or attempted to suckle her babe.

On the fourth day, October 26th, her pulse was frequent; the breasts were much swelled, and the axillary glands painful; she had a little head-ach, and felt very languid. The lochial discharge was pretty great. Several clysters remained without effect, but the urinary discharge was voluntary.

On the fifth day, she was in general much the same, the head-ach only somewhat worse, and the lochia had almost entirely disappeared; but the milk flowed abundantly and the swelling of the breasts had decreased. The clysters, even those of a more stimulating nature, had remained mained without effect; in consequence of which, Mr. G. ordered the patient to take a tea-spoonful of lenitive electuary, with mucilaginous drink acidulated with a little lemon-juice, keeping herself as quiet as possible.

On the sixth day, the electuary had procured an evacuation during the night; the fæces were passed with much pain, causing a great deal of florid blood to flow from the vagina, however this ceased on her lying quiet. She complained of a kind of burning sensation she had felt in the abdomen during the night, and which still continued; a little blood resembling the lochiæ re-appeared; the head-ach had gone off on the bowels being moved. She had more appetite, showed particular inclination for beer; and she was permitted to take a glass of white beer.

On the seventh day, she was in general tolerably well; the pulse being soft and slow, and the burning sensation in the abdomen having ceased. But now the infant, which hitherto had been quite well, was suddenly taken ill, with periodical symptoms of suffocation, rattling in the throat for a whole hour together, and sudden changes of complexion. An emetic in the form of a linctus was prescribed, together with a clyster and an aromatic bath in the evening.

On the night of the eighth day, (on the 30th of October,) the child appeared quite easy, sucked, and was apparently quite well; but, in the afternoon, by four o'clock, it was suddenly taken with spasms, which, after intermitting one hour, re-appeared, and the child thus died with general cataleptic symptoms. It was a boy, to all appearance of a healthy and firm constitution, weighing between seven and eight pounds. Dissection was not permitted.

The infant's death much agitated the mother, for whom, though continuing tolerably well till the evening of the 31st, Mr. G. apprehended some ill consequences; and, indeed, on the first of November, vestiges of an increased action of the vessels of the wounds of the womb and the vagina, made their appearance, an increased discharge of a purulent serum taking place; the patient complaining of pressure in the abdomen and the bottom of the pelvis, which sensation continued, but without increasing till the 2d of November. Her pulse was quick and full, and a certain degree of inflammation was apparent.

An antiphlogistic regimen, with an injection of a decoction of herba hyosciami, repeated three or four times, procured relief; so that, on the 5th of November, and the following days, she felt as usual, her pulse being again soft and composed. She now complained only of being languid, and

 sometimes sometimes of a painful sensation in the lower extremities, and anxiety towards night lasting above an hour at the time. Her bowels being however regular, and her appetite good, Mr. G. thought proper to await the further issue, till the 9th of November, when he prescribed her a mixture of Infus. Valerianæ, Extr. Trifol. fibrin. Liquor Ammon. Succin. Tincture of Opium, and some syrup, which at once dispersed those febrile symptoms. Every examination of the internal pudenda had hitherto been made without any decisive result, as they always caused great pain, and the margins of the artificial os tincæ, formed by the incisions, feeling like irregular fleshy tumors. An inclination to an erysipelatous inflammation was very evident in these parts, on account of which, a solution of sugar of lead with opium was applied.

On the 12th of November, the twentieth day after the operation, the edges of the wound were no longer swelled; the small incisions had lately disappeared; and, in that place where the natural os tincæ ought to have been situated, was a large transverse fissure of about one inch, the anterior labium of which was little thicker than the posterior. This day the patient left her bed for the first time without that sensation which, for some days before, she had described, not indeed as a pain, but rather as a certain pressure, producing a sensation as if something was about to drop from the external genitalia.

The patient continued perfectly well till the twenty-fourth day, (the 16th of November). On the twenty-fifth, she was again taken with fever and inclination to vomit, violent abdominal pains, particularly in the sides, accompanied with obstinate costiveness, a discharge of florid blood having also re-appeared several days before. Surfeits and exposure to cold were the causes of these symptoms, which continued for about ten days. A few tamarind mixtures, however, relieved the costiveness, and with it, the spasmodic symptoms; but the greatest service was derived from an emetic, given one of the last days, followed by a mixture of cinnamon-water, with some stimulants added to it. Externally, an embrocation of Peruvian balsam, with spirits of soap, was rubbed in the abdominal integuments, which finally removed every uneasiness, and the patient has continued quite well ever since.

*Hamburgh; March* 21, 1817.

